# A streamlined pipeline based on HmmUFOtu for microbial community profiling using 16S rRNA amplicon sequencing

**DOI:** 10.5808/gi.23044

**Published:** 2023-07-31

**Authors:** Hyeonwoo Kim, Jiwon Kim, Ji Won Choi, Kwang-Sung Ahn, Dong-Il Park, Sangsoo Kim

**Affiliations:** 1 Department of Bioinformatics, Soongsil University, Seoul 06978, Korea; 2 Department of Biological Sciences, Sungkyunkwan University, Suwon 16419, Korea; 3 Functional Genome Institute, PDXen Biosystems, Co., Daejeon 34027, Korea; 4 Division of Gastroenterology, Department of Internal Medicine and Inflammatory Bowel Disease Center, Kangbuk Samsung Hospital, Sungkyunkwan University School of Medicine, Seoul 03181, Korea; 5 Medical Research Institute, Kangbuk Samsung Hospital, Sungkyunkwan University School of Medicine, Seoul 03181, Korea

**Keywords:** amplicon sequencing, DADA2, HmmUFOtu, metagenomics

## Abstract

Microbial community profiling using 16S rRNA amplicon sequencing allows for taxonomic characterization of diverse microorganisms. While amplicon sequence variant (ASV) methods are increasingly favored for their fine-grained resolution of sequence variants, they often discard substantial portions of sequencing reads during quality control, particularly in datasets with large number samples. We present a streamlined pipeline that integrates FastP for read trimming, HmmUFOtu for operational taxonomic units (OTU) clustering, Vsearch for chimera checking, and Kraken2 for taxonomic assignment. To assess the pipeline’s performance, we reprocessed two published stool datasets of normal Korean populations: one with 890 and the other with 1,462 independent samples. In the first dataset, HmmUFOtu retained 93.2% of over 104 million read pairs after quality trimming, discarding chimeric or unclassifiable reads, while DADA2, a commonly used ASV method, retained only 44.6% of the reads. Nonetheless, both methods yielded qualitatively similar β-diversity plots. For the second dataset, HmmUFOtu retained 89.2% of read pairs, while DADA2 retained a mere 18.4% of the reads. HmmUFOtu, being a closed-reference clustering method, facilitates merging separately processed datasets, with shared OTUs between the two datasets exhibiting a correlation coefficient of 0.92 in total abundance (log scale). While the first two dimensions of the β-diversity plot exhibited a cohesive mixture of the two datasets, the third dimension revealed the presence of a batch effect. Our comparative evaluation of ASV and OTU methods within this streamlined pipeline provides valuable insights into their performance when processing large-scale microbial 16S rRNA amplicon sequencing data. The strengths of HmmUFOtu and its potential for dataset merging are highlighted.

## Introduction

16S rRNA amplicon sequencing, facilitated by the next-generation sequencing technology has revolutionized the microbial community profiling [1]. The typical workflow involves polymerase chain reaction amplification targeting the hypervariable V3–V4 or V4 region of the microbial 16S small subunit of rRNA, followed by paired-end sequencing (250 or 300 bp) of the amplicons using the Illumina MiSeq platform. The resulting sequenced reads are commonly trimmed at a preset quality threshold due to decreasing sequence quality toward 3′-end, inherent in Illumina sequencing-by-synthesis technology. The trimmed paired-end reads are then assembled into contigs representing the amplicon sequences.

Excessive trimming at 3′-end can pose challenges particularly in V3–V4 sequencing, as the paired reads may not overlap and fail to form a contig. This issue arises because, given 2 × 300 bp sequencing, the pair can overlap for a maximum of 150 bp. Traditionally, a data processing pipeline involves *de novo* clustering of quality-controlled contigs into operational taxonomic units (OTUs) at a 97% identify threshold, providing a genus-level resolution. However, *de novo* OTU clustering becomes increasingly challenging as the number of contig sequences grows, especially when handling thousands or more samples.

Furthermore, performing *de novo* OTU clustering requires processing contig sequences from all the samples together, making it tempting to split the dataset into smaller batches. However, merging the clustering results from the split batches is not straightforward. Closed-reference OTU clustering offers a solution to this problem. It involves mapping sample contigs to pre-existing OTUs generated from reference sequences, typically at a 97% identity threshold. This method enables easy merging of clustering results from different batches, facilitating the processing of large-scale datasets. Nonetheless, closed-reference OTU clustering has a drawback in cases where the sample taxa are not represented in the reference dataset. Such sequences cannot be reliably assigned to any OTUs and are usually discarded.

HmmUFOtu is one of the closed-reference OTU clustering methods that utilize a phylogenetic tree constructed from reference sequences [2]. This tree is pruned at a specified identity level (usually 97%), resulting in a set of OTUs comprising both the reference sequences and internal nodes representing common ancestors. Each sample contig sequence is then phylogenetically placed within the tree and assigned to the closest OTU. Even if the taxon of the sample contig is not present in the reference sequence set, it can still be captured by one of the internal node OTUs.

One advantage of HmmUFOtu is that it does not rely on overlap between the paired-end sequences for contig assembly. Instead, it employs a hidden Markov model (HMM) method to independently map forward and reverse reads to a pre-aligned set of reference sequences. This approach allows contigs to be assembled even when excessive trimming at the 3′-ends eliminates overlapping regions, with defined gaps included.

While HmmUFOtu offers several benefits, such as increased contig assembly flexibility, it may generate a large number of OTUs compared to the expected number of taxa in a typical sample set. In fact, the number of OTUs produced by HmmUFOtu can be tens of thousands, potentially twice as many as expected. Additionally, it is worth noting that the chimera checking in HmmUFOtu is limited to the reference sequences, which means some real chimeras may not be detected.

Recently, amplicon sequence variant (ASV) methods have been introduced [3-5] and are widely used. In various benchmarking tests, it outperformed OTU clustering methods by yielding high-resolution taxonomic profiling. However, ASV methods rely on sufficient overlap between forward and reverse reads, which can lead to the discarding of a significant number of read pairs when excessive trimming is required, particularly with V3–V4 amplicons. For example, some community (https://forum.qiime2.org/t/dada2-too-much-reads-discard-after-dada2/20389, https://github.com/benjjneb/dada2/issues/1133) users experienced more than half of the contigs discarded. When a large number of input sequences are discarded and do not contribute to abundance profiling, downstream analyses based on the abundance profile require cautious interpretation.

Here we report a pipeline built around HmmUFOtu by incorporating quality control and chimera checking as well as phylotyping, all based on publicly available software. In order to demonstrate the performance of the pipeline, 16S rRNA V3–V4 amplicon sequences of two independent stool datasets from normal Korean participants were processed with our pipeline and the results are reported. Additionally, we processed the same datasets with one of the popular ASV methods, DADA2 [5], for comparison.

## Methods

### 16S rRNA amplicon sequencing datasets

We used two published 16S rRNA V3–V4 amplicon sequencing datasets. They were generated from the stool samples of independent normal Korean cohorts recruited at the corresponding healthcare centers. One dataset was downloaded from the EBI ENA database (accession ERP116736) [6]. It consisted of 890 MiSeq paired-end fastq files, initially containing 125,822,144 read pairs. However, it was noticed that some fastq files were duplicated and a total of 104,443,918 read pairs were retained after the deduplication. The other dataset was acquired through the communication with the authors [7]. It comprised 1,462 MiSeq paired-end fastq files with a total of 58,631,215 read pairs. We designate this dataset as “SMK”.

### Sequence quality control and trimming

We employed Fastp version 0.23.2 [8] for quality control of the 300 bp × 2 next-generation sequencing amplicon paired-end reads. Both the forward and reverse reads of an amplicon were screened for low-quality reads, with a mean Phred score threshold of 20. Any reads failing below this threshold were trimmed at the 3′-end. Additionally, reads shorter than 150 bp were excluded from further analysis. If one of the pairs did not pass the quality control criteria, both reads were removed, ensuring each amplicon is represented by paired-end reads.

### HmmUFOtu reference rebuilding

The source code of HmmUFOtu (version 1.5.1, https://github.com/Grice-Lab/HmmUFOtu) was cloned from GitHub and compiled according to the instruction. Additionally, we downloaded the pre-built reference multiple alignment and phylogenetic tree from the HmmUFOtu website. These references were based on the GreenGene (version 13.8, https://upenn.box.com/shared/static/o146rpg53ebmn3pxikf7zm1uwatez6sl.zip) sequence collection, which consists of 198,643 sequences. Among these sequences, 99,322 represent the actual sequences corresponding to the tips of the phylogenetic tree, while the remaining sequences correspond to the inferred internal nodes. To enhance the reference collection, we augmented it with the RDP Classifier Training Set (version 18, https://sourceforge.net/projects/rdp-classifier/files/RDP_Classifier_TrainingData), which added a total of 21,195 sequences.

To align the 21,195 RDP Training Set sequences with the multiple alignment of the 99,322 GreenGene tip sequences, we utilized the Infernal version 1.1.4 package. After visual inspection of the multiple alignment, we identified and removed four obvious outlier sequences. Additionally, positions where more than 99% of the sequences have gaps were excised from the alignment. To further refine the multiple alignment, we employed the AdjustAlignment function of the DECIPHER Bioconductor package.

Next, we constructed a phylogenetic tree using FastTree with the GTR model. A total of 114 tips having 0 branch lengths were removed from the tree, resulting in a final tree with 240,804 nodes. Finally, we utilized the HmmUFOtu-build program to build the HmmUFOtu model.

### HmmUFOtu clustering and chimera removal

After the Fastp trimming process, each pair of the 16S rRNA V3–V4 amplicon fastq files was aligned to the HmmUFOtu model. This alignment produced an aligned contig sequence for each pair. The contig was then placed onto the reference phylogenetic tree and assigned to the nearest node using the HmmUFOtu main program.

The amplicons associated with a common HmmUFOtu node were gathered, and their consensus sequences were generated using the Biostrings Bioconductor package. This process was performed for each individual sample. Subsequently, the size of each cluster was compiled from all the samples, resulting in the creation of an abundance table.

To identify and remove chimeric sequences within the consensus sequences, we utilized the *de novo* chimera checking command in the Mothur version 1.48 program. Each consensus sequence was distributed to its corresponding samples and assessed for potential chimeric combinations of more abundant sequences within that specific sample. Following the guidelines provided by the developer of Mothur, we removed the chimeric sequences only in the samples where the chimerism was detected.

### ASV clustering using DADA2

The raw sequences were subjected to trimming using Fastp, resulting in the removal of bases from the tails and producing sequences of 290 bp length. No quality filtering was applied during this trimming process. Subsequently, Figaro [9] was employed to identify the optimal trimming end positions. The parameters “trimPosition” and “maxExpectedError” were adjusted to achieve the highest “readRetentionPercent” value. The optimized parameters were then utilized in the subsequent DADA2 run, version 1.14.1, installed from Bioconductor. Following the tutorial process given on the project website (https://benjjneb.github.io/dada2/tutorial.html), DADA2 was employed to cluster the sequences into ASVs.

### Taxonomy assignment and phylotyping

We employed Kraken2 version 2.1.2 [10] to perform taxonomic classification of the sequences. The reference sequence database used for classification was downloaded from EzBioCloud (version 2019, https://www.ezbiocloud.net/resources/16s_download) and contained a total of 64,660 sequences with taxonomic information down to the species level. This reference database was processed to construct the Kraken2 16S rRNA database. Following classification of the OTUs or ASVs using Kraken2, the taxonomy information was reduced to the genus level. Subsequently, a custom R script was utilized to generate a phylotype abundance table by enumerating the unique trimmed taxa.

### β-diversity calculation and principal coordinates analysis plotting

β-diversity was assessed using two dissimilarity measures: Jaccard dissimilarity and Yeung-Clayton’s θ dissimilarity. Jaccard dissimilarity considers only the presence or absence of a taxon, disregarding its abundance. On the other hand, Yeung-Clayton’s θ dissimilarity takes into account differences in taxon abundance.

To account for variation in total abundance across samples, subsampling was performed based on the least abundant sample from the phylotype abundance table. This subsampling process was repeated randomly 1,000 times, and the averaged dissimilarity indices were calculated. Principal coordinate analysis was then employed to perform ordination using these averaged dissimilarity indices. All these computations were carried out using Mothur (version 1.48) [11].

### Sample clustering using Dirichlet multinomial modeling

The phylotype abundance table was subjected to clustering using Dirichlet multinomial mixture (DMM) modeling. This approach aims to identify the optimal combination of the representative abundance profiles that can describe the overall abundance table. By scanning a specified range of cluster numbers, DMM seeks to find the best solution. The clustering of samples is performed using the maximum a posterior estimate.

To perform DMM clustering, the “communitytype” command in Mothur was utilized. This command facilitated the calculation of DMM and enabled the determination of clusters based on the abundance profiles in the phylotype abundance table.

### Availability

The bash, perl, and R scripts developed in the current study are available through GitHub (https://github.com/sskimb/SSU-microbiome).

## Results

### Overview of the pipeline

**Fig. 1.**
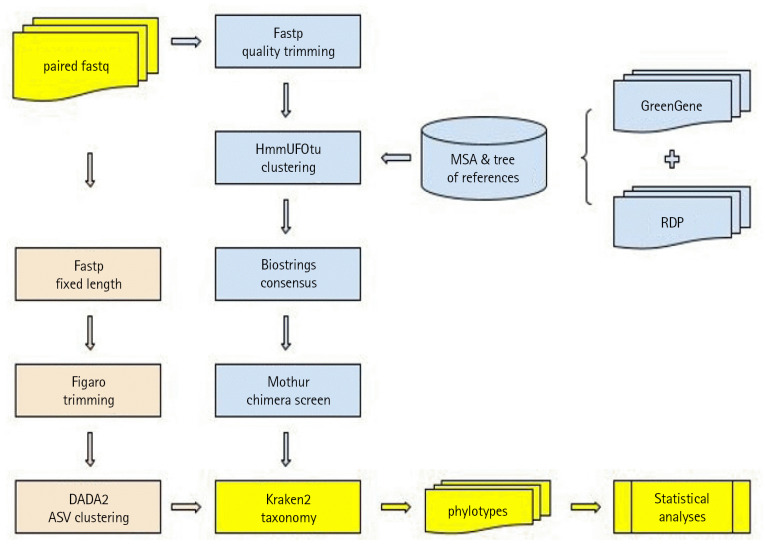
The outline of the pipeline. The objects filled by light blue represent the pipeline that includes HmmUFOtu clustering as proposed in this work. The objects filled by apricot represent the steps taken with DADA2 for benchmark comparison. The yellow objects represent the input and output items as well as common analyses steps.

The pipeline proposed in this study is outlined in Fig. 1. The input consists of quality trimmed paired-end fastq files, which are then subjected to phylogenetic OTU clustering using HmmUFOtu. The process utilizes an HMM that represents the multiple alignment of the reference sequences, as well as a phylogenetic tree of these sequences.

From the HmmUFOtu clustering result, consensus sequences are generated for each cluster using the Biostrings package in Bioconductor. These consensus sequences undergo a screening step to detect chimeric combinations, which is performed using the *de novo* chimera checking algorithm in Mothur. The taxonomic annotations of the non-chimeric clusters are obtained using Kraken2.

Next, clusters assigned to the same taxonomic groups are merged to form phylotypes using a custom R script. Phylotypes represent higher-level taxonomic units that encompass multiple closely related clusters. This merging step helps to reduce the complexity of the data and provides a more consolidated view of the microbial community composition. Once the resulting phylotype abundance table is prepared, downstream statistical analyses can be performed using Mothur, a versatile software package that offers a range of statistical tools for analyzing microbial community data.

For benchmarking processes, the same paired-end fastq files are processed using the DADA2 protocol, available as a Bioconductor package. The output from DADA2, consisting of ASVs, is also taxonomically annotated using Kraken2 and merged into phylotypes as described above.

### Benchmark results of the HmmUFOtu clustering and DADA2 pipeline using ERP116736

Both the ERP116736 and SMK datasets were processed using both the DADA2 and HmmUFOtu pipelines. For the ERP116736 dataset, both pipelines initially started with 890 paired-end fastq files with a total of 104,443,918 read pairs (Table 1). However, the DADA2 pipeline experienced a significant loss of read pairs, with more than half being discarded after denoising and chimera removal steps. In contrast, the HmmUFOtu pipeline retained approximately 93% of the total read pairs.

Each ASV or OTU generated by both pipelines was taxonomically classified using Kraken2 and the EzBioCloud taxonomy database (version 2019). ASVs/OTUs belonging to the same taxonomic group were merged, and their abundances were summed. As a post-processing step, phylotypes observed fewer than 10 times across all samples were removed to reduce noise. The HmmUFOtu pipeline generated several times more phylotypes compared to DADA2.

Additionally, any samples with a total abundance less than 20,000 were excluded from the subsequent analyses. In the cases of the DADA2 pipeline, 35 samples did not meet this threshold, while none of the samples processed with the HmmUFOtu pipeline were excluded. However, the filtering step following phylotyping only resulted in a small fraction of the total abundance being lost in either case.

**Fig. 2.**
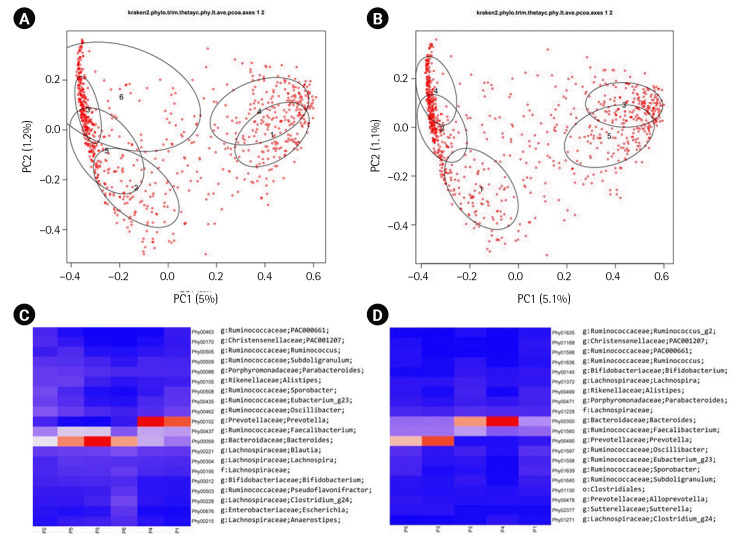
β-diversity plots and the corresponding heatmaps. The first two dimensions of the principal coordinate analyses of β-diversity based on Yeung-Clayton’s θ are shown for ERP116736 (A) and SMK (B). Dirichlet multinomial mixture models (DMM) were fitted to the abundance tables, resulting in 6 and 5 clusters for ERP116736 and SMK, respectively. The ellipses are centered at the center-of-mass points (numbered 1 to 6 and 1 to 5 for ERP116736 and SMK, respectively) of the samples belonging to each DMM cluster and drawn at 50% confidence level. The percent relative abundance of each phylotype in each cluster, as inferred from the modeling, are represented as heatmaps for ERP116736 (C) and SMK (D). In the heatmaps, the cluster numbers are prefixed with ‘P’. Red and blue depict higher and low abundance, respectively, while orange and white depict intermediate abundance.

The original publication of ERP116736 [6] reported the presence of two major enterotypes [12] in its β-diversity plot manifested. These enterotypes were characterized by a dominance of either the *Bacteroides* genus or the *Prevotella* genus. In our analysis, we calculated the β-diversity of each dataset using Yeung-Clayton’s θ dissimilarity and visualized the result through Principal Coordinate Analysis (Fig. 2A and 2B). Additionally, we employed DMM models to cluster the samples based on their abundance profiles. The resulting mean phylotype profiles for each cluster were represented as heatmaps in Fig. 2C and 2D. When overlaying the ellipses indicating the cluster membership onto the β-diversity plots, we observed that the clusters could be grouped into two categories for both the DADA2 and HmmUFOtu pipelines. In the DADA2 result, clusters 1 and 4 formed one group, while clusters 2, 3, 5, and 6 formed the other group. Similarly, in the HmmUFOtu result, clusters 3 and 5 formed one group, while clusters 1, 2, and 4 formed the other group. The corresponding heatmaps of the top 20 phylotypes also displayed clear differentiation between the two groups. These findings are consistent with the original publication, highlighting the presence of two major groups in the ERP116736 dataset, with one group dominated by Prevotella and the other by Bacteroides.

### Benchmark results of the HmmUFOtu clustering and DADA2 pipeline using SMK

In contrast to the ERP116736 dataset, the results obtained with the SMK dataset showed significant differences (Table 2). This SMK dataset comprised 1,462 paired-end fastq files with a total of 58,631,215 read pairs. However, the DADA2 pipeline experienced a loss of more than 80% of the read pairs during denoising and chimera removal steps. As a result, the phylotyping and filtering processes were ineffective, with only 34 samples surviving, accounting for a small fraction of the original read pairs. In contrast, the HmmUFOtu pipeline retained approximately 89% of the total read pairs, allowing for the successful phylotyping and filtering of 1,070 out of 1,462 samples. These findings suggest that HmmUFOtu demonstrated robustness in handling this seemingly noisy SMK dataset. Despite the SMK dataset having more samples than ERP116736, the total number of read pairs was considerably smaller. Nonetheless, the number of OTUs or phylotypes identified by HmmUFOtu did not differ significantly between these two datasets.

### Merging the datasets ERP116736 and SMK

**Fig. 3.**
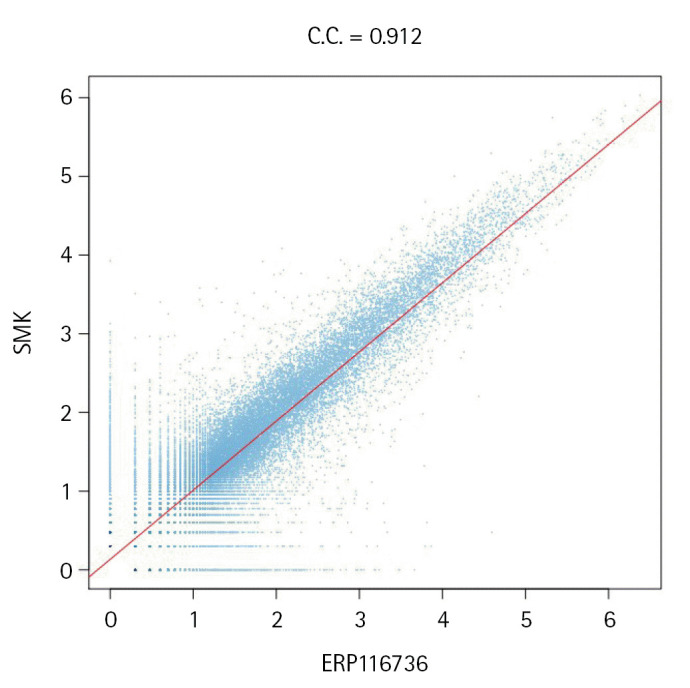
Scatter plot of the total abundance of each operational taxonomic units common to both datasets. The X and Y axes are in log10-scale after adding 1 as pseudo-count.

One of the advantages of closed-reference OTU clustering is the ability to merge independently processed datasets. In our study, both the ERP116736 and SMK datasets were obtained from the cohorts with similar ethnicity and region, although they were collected by different healthcare centers and sequenced by different service providers using a similar protocol. We aimed to compare the total abundance of each OTU identified by the HmmUFOtu pipeline, which resulted in 41,398 and 42,657 denoised and chimera removed OTUs for ERP116736 and SMK, respectively. By merging these OTU sets, we obtained a total of 51,597 OTUs, of which 32,458 OTUs were common to both datasets. Fig. 3 demonstrates that the total abundances of these shared OTUs were highly similar, with correlation coefficients of 0.902 and 0.919 in linear and logarithmic scales, respectively. This indicates a consistent and comparable representation of the common OTUs across the datasets.

The merged dataset was further processed by converting it into phylotypes, and DMM models were applied to the abundance matrix. The optimal model identified in this analysis consisted of seven clusters, which were labeled P1 through P7. Table 3 provides information on the distribution of samples from each dataset among these clusters, revealing a non-homogeneous partitioning of the datasets (p_chisq_ < 2.2 × 10^-16^). Some clusters showed an enrichment of samples from the ERP116736 dataset, while other clusters displayed an enrichment of samples from the SMK dataset. This suggests distinct patterns and composition within the clusters, with certain clusters being more prevalent in one dataset compared to the other (Table 3).

**Fig. 4.**
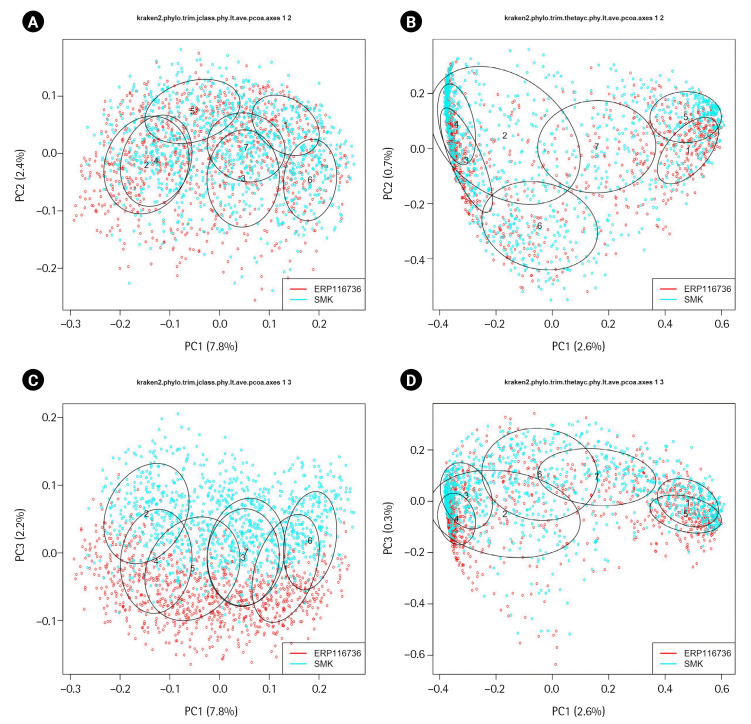
β-diversity plots of the merged dataset. Dissimilarity indices were Jaccard coefficients (A, C) and Yeung-Clayton’s θ (B, D). The first two dimensions of the principal coordinate analysis were shown in the Fig. 4A and 4B and the first and third dimensions were displayed in the Fig. 4C and 4D. The ellipses representing the clusters of the Dirichlet multinomial mixture modeling, were centered at the center of mass and drawn at 50% confidence levels.

The β-diversity plot in Fig. 4. revealed that the samples from both ERP116736 and SMK datasets exhibited mixing in the first two dimensions. However, the third dimension showed a clear segregation between the datasets, indicating distinct compositional differences. The segregation was more pronounced in the Jaccard dissimilarity metric compared to Yeung-Clayton’s θ dissimilarity, suggesting that the presence or absence of certain phylotypes in one dataset relative to the other might be responsible for the observed segregation.

**Fig. 5.**
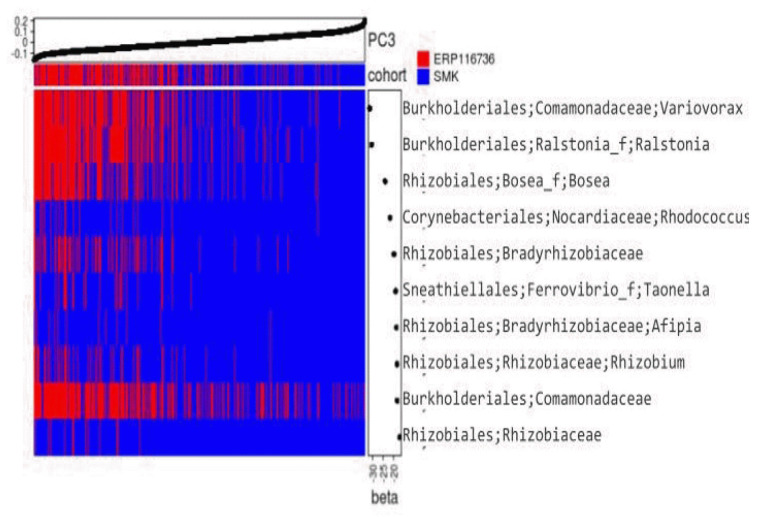
Heatmap of top 10 phylotypes that segregate the datasets. The heatmap was colored red and blue to depict presence and absence, respectively, of the phylotype in each sample. The columns representing samples were ordered in ascending order of PC3 of the principal coordinates analysis based on Jaccard coefficient (y-axis of Fig. 4C) as shown at the upper panel. The rows representing phylotypes were ordered in ascending order of the beta coefficient of the logistic regression that fits the presence or absence of a phylotype by PC3. Taxonomy of each phylotype is based on EzBioCloud 2019 and abbreviated at the order to genus level, delimited by semicolon.

To investigate this further, logistic regression was employed using the PC3 of Jaccard dissimilarity as the predictor for classifying the phylotypes into presence or absence. Among the phylotypes that exhibited a statistically significant fit (p < 10^-5^), top 10 phylotypes with the largest effect size (beta coefficient) are shown in Fig. 5. Interestingly, all of these top 10 phylotypes displayed negative beta coefficients, indicating that they were more frequently observed in the ERP116736 dataset compared to the SMK dataset. This observation aligns with the heatmap analysis, supporting the distinct compositional differences between the datasets.

Notably, it is worth mentioning that the *Comamonadaceae* family and *Ralstonia* genus, which are among the top 10 phylotypes with significant effect sizes, have been previously reported as contaminants in some stool samples [13,14]. This finding adds further support to the notion that these phylotypes may contribute to the segregation observed between the ERP116736 and SMK datasets.

This section should describe the results of the experiments. Extensive interpretation should be reserved for the Discussion section. The results should be presented as concisely as possible. Footnotes should not be used and will be transferred to the text. Gene symbols should be italicized; protein products are not italicized.

## Discussion

In our study, we observed that the DADA2 pipeline, which utilizes the ASV method, resulted in the discarding of a significant portion of the original read pairs during the denoising and chimera removal steps. This led to concerns regarding the comparability of abundances between taxa and the need for cautious interpretation of downstream analyses based on abundance data. Our reprocessing of two published datasets, ERP116736 and SMK, using the DADA2 pipeline revealed substantial losses in read pairs, with ERP116736 losing over 55% and SMK losing over 80% of the total read pairs.

Notably, the SMK dataset appeared to be of lower quality compared to ERP116736, as indicated by the higher percentage of read pair losses in the DADA2 pipeline. This quality issue became more evident during the subsequent phylotyping and filtering steps, where a large number of samples from SMK had to be excluded due to low total abundance. In contrast, ERP116736 exhibited a much smaller loss of samples due to low total abundance. Unfortunately, the original publication of ERP116736 did not provide detailed processing statistics, making it difficult to directly compare our findings with theirs. However, our adherence to the recommended processing steps by the developers suggests that a loss of approximately 55% of total read pairs when using DADA2 is not uncommon.

In our study, we observed that our streamlined pipeline based on HmmUFOtu was able to preserve the majority of the total read pairs, losing only a small percentage for both the ERP116736 and SMK datasets. This is in stark contrast to the DADA2 pipeline, which resulted in substantial read pair loss, particularly for the SMK dataset, indicating its lower quality compared to ERP116736. Interestingly, despite the significant differences in read pairs retention, the global patterns of β-diversity plots generated by DADA2 and our pipelines for ERP116736 were similar. This suggests that the DADA2 quality control step may have uniformly removed read pairs across the taxa in a high-quality dataset like ERP116736.

Furthermore, while the DADA2 pipeline produced unusable results for downstream analyses with the SMK dataset, our pipeline based on HmmUFOtu retained a large proportion of the read pairs and samples, yielding a comparable number of OTUs or phylotypes to ERP116736. This demonstrates the robustness of our pipeline in handling the seemingly noisy SMK dataset. Moreover, the successful merging of ERP116736 and SMK datasets, along with the high correlation in OTU abundance, indicates that despite being collected from different healthcare centers and sequenced by different service providers, the main features of these datasets are similar.

We also examined the β-diversity principal coordinates analysis (PCoA) plot based on different metrics, namely Yeung-Clayton's θ and Jaccard dissimilarity. The PCoA plot based on θ revealed clear separation of the two enterotypes reported in the original study of ERP116736, characterized by dominance of Bacteroides and Prevotella. However, the PCoA plot based on Jaccard dissimilarity displayed the DMM clusters positioned nearby without clear separations. This suggests that the differences between DMM clusters in terms of Jaccard dissimilarity may not be significant enough to distinctly separate them, compared to the separation observed with θ. It implies that the enterotypes may differ more in terms of abundance profiles rather than taxonomic variety. It is worth noting that enterotypes, which are correlated with long-term dietary habits, can potentially be altered by changes in diet. However, longitudinal studies have shown that the transition from one enterotype to another is rare, possibly due to ecological barriers [15].

The ability to merge independently processed datasets is indeed a valuable advantage of closed-reference OTU clustering methods like HmmUFOtu. By utilizing this approach, it becomes feasible to create a comprehensive database that encompasses numerous datasets, all standardized by HmmUFOtu's OTUs. This compilation of datasets provides a valuable resource for conducting comparative studies with ease.

One of the notable strengths of HmmUFOtu is its ability to process samples in a sample-by-sample manner. This feature allows for concurrent processing of multiple samples, taking advantage of available computing nodes. By efficiently utilizing the computational resources, HmmUFOtu enables the analysis of a large number of samples in a timely manner. This scalability makes it possible to handle extensive datasets and accelerates the pace of research.

Merging datasets at the phylotype level can be a useful strategy for comparative studies. However, it is important to consider certain factors when utilizing this approach. One challenge arises from the dependency on the taxonomy reference dataset, which requires updating the phylotyping process whenever the reference dataset is updated. This means that each time a new taxonomy reference dataset is released, the phylotyping process needs to be repeated to ensure consistency and accuracy.

In contrast, HmmUFOtu is less impacted by updates to the taxonomy reference dataset. This is because the OTU clustering performed by HmmUFOtu is based on the phylogenetic structure of the reference sequences rather than relying solely on taxonomic information. Consequently, if a novel microorganism is discovered in a sample and it is distinct from the species in HmmUFOtu's reference dataset, it would be captured by one of the internal nodes of the reference phylogenetic tree. This inherent flexibility of HmmUFOtu allows for the detection and inclusion of novel taxa without requiring immediate updates to the reference dataset.

However, it is crucial to be cautious when interpreting the results obtained from HmmUFOtu, as there is a potential for OTUs assigned to internal nodes to represent multiple distinct taxa. When encountering phylotypes corresponding to internal nodes, it becomes necessary to conduct further examination and analysis to assess the similarity and cohesiveness of the member sequences within the assigned group.

In conclusion, our comparison of the DADA2 and HmmUFOtu pipelines on two datasets revealed that HmmUFOtu retained a higher proportion of read pairs and samples, making it more robust for analyzing low-quality datasets. The ability to merge independently processed datasets using HmmUFOtu offers the potential for creating a comprehensive database for comparative studies.

## Figures and Tables

**Table 1.** Benchemark results using the dataset ERP116736

Category	Item	Pipeline	
		DADA2	HmmUFOtu
Denoised	ASVs/OTUs	661,519	47,408
	Total read pairs	74,046,442	101,861,374
Chimera removed	ASVs/OTUs	71,338	43,824
	Total read pairs	47,117,364	97,381,765
Phylotyping with filtering (abundance > 20,000 & count >10)	Samples	855	890
	Phylotypes	574	1,805
	Total abundance	46,552,783	97,374,156

ASV, amplicon sequence variant; OTU, operational taxonomic units.

**Table 2.** Benchemark results using the dataset SMK

Category	Item	Pipeline	
		DADA2	HmmUFOtu
Denoised	ASVs/OTUs	148,363	48,219
	Total read pairs	14,139,973	55,684,680
Chimera removed	ASVs/OTUs	35,180	42,657
	Total read pairs	10,813,664	52,286,048
Phylotyping with filtering (abundance > 20,000 & count > 10)	Samples	34	1,070
	Phylotypes	156	1,711
	Total abundance	840,926	47,312,617

ASV, amplicon sequence variant; OTU, operational taxonomic units.

**Table 3.** Number of samples partitioned into each cluster of the Dirichlet multinomial mixture modeling of the merged dataset

Dataset	P1	P2	P3	P4	P5	P6	P7
ERP116736	150	85	134	235	131	76	78
SMK	90	172	193	151	144	151	168
